# Analysis of developmental trends in physical activity, BMI and muscles in children and adolescents with mild-to-moderate intellectual disability

**DOI:** 10.1016/j.heliyon.2021.e07457

**Published:** 2021-07-03

**Authors:** Jitka Kampasová, Hana Válková

**Affiliations:** Masaryk University, Faculty of Sports Studies, Department of Social Sciences and Sports Management, Kamenice 5, Brno 625 00, Czech Republic

**Keywords:** Intellectual disability, Children, Adolescents, Pupils, Physical activity, BMI, Muscles, Postural balance, Movbands, Trends

## Abstract

**Introduction:**

There is a lack of longitudinal research of physical activity (PA) of pupils with intellectual disability (ID). The aim of the research was to identify the trends in PA, BMI and muscles in children and adolescents with ID in the Czech Republic over 2-year period and find whether 2-month summer holidays have effect on developmental trends. The aim was also to find out the level of PA of children and its correlation with BMI and muscles and the correlation between postural balance and children's muscles.

**Methods:**

Participants were pupils aged 8–19 (in each measurement was n = 23, n = 37, n = 36, n = 33). They wore a movband for 1 week. InBody analyser and a test of Single leg stance with eyes open were used.

**Results:**

The trend of PA in girls and boys is convex and the trend of their BMI is unbalanced. In pupils with mild and moderate ID, the trend of PA, the trend of BMI and the trend of muscles is unbalanced. Summer holidays cause a decrease in BMI values for all categories. In pupils with moderate ID, summer holidays cause an increase in PA and an increase in muscle (both statistically significant). The correlation between PA and muscle and BMI is ambiguous. Also, the correlation between postural balance and muscles is ambiguous.

**Conclusions:**

Children's PA reaches 74–122% of the norm. Girls, boys, pupils with mild and moderate ID have normal weight. In the Czech Republic children with ID have many opportunities to participate in sport events during the year.

## 1 Introduction

Physical activity (PA) is associated with both physical and mental health benefits (increased self-esteem, decreased depression) in children and adolescents (Janssen and LeBlanc, 2010; Biddle and Asare 2011). Exercise is beneficial for all age groups (Kalman et al., 2009, p. 22). Aerobic activities have the greatest health benefits (Janssen and LeBlanc, 2010).

Due to the adaptation of the body to exercise, there is, for example, a reduction in cholesterol, a decrease in the heart rate (the heart works more efficiently) and blood pressure, higher oxygen levels are transported from the blood to the muscles, tendons and bones are strengthened, muscle mass increased, muscle coordination is improved. Exercise also increases self-confidence, and helps to compensate for the stress of everyday life, etc. (Jančík et al., 2006; Fischer and Secher, 2019) Children with ID who do sports have healthier cardiovascular profiles, are slimmer, have more bone mass, which reduces the risk of osteoporosis at the old age (Boreham and Riddoch, 2010).

It is well known that people with disabilities have more health problems than people without disabilities (Pančocha, 2006, p. 83). They have more motor difficulties and more health issues, such as epilepsy, cerebral palsy, Down's syndrome, autism, anxiety, etc. (Oeseburg et al., 2011; Aljerf and Aljurf, 2020), low physical fitness and a high prevalence of obesity (Salaun and Berthouze-Aranda, 2012; Hartman et al., 2015). People with Down syndrome have fitness levels and worse coordination (Guistino et al., 2021). People with ID have lower motor skills. There is also a strong correlation between motor skills and language skills (Houwen et al., 2016). The positive effects of PA are far more important for children and adolescents with intellectual disability than for their peers. Disabilities in connection with unhealthy lifestyle often lead to increased health risks (Pančocha, 2006, p.83). Increasing physical activity could help to reduce these risks and the consequent health inequality (Maiano, 2010).

Furthermore, there is a direct link into adulthood, in which the good health of adults results from their good health in their childhood. Behavior is also transmitted, with physically active children likely to become physically active adults (Boreham and Riddoch, 2010). People with ID are at risk of weight gain in the period of transition from the adolescence into adulthood (Mitchel et al., 2016).

Children's physical activity is also essential for acquiring motor skills. This fact is often overlooked (Loprinzi et al., 2017; Stodden et al., 2008). Physical education develops mental functions such as perception, observation, memory, attention, imagination, thinking and speech (Kvapilík and Černá, 1990, p. 91). The development of physical activities needs to be addressed responsibly for people with intellectual disability throughout their lives and especially in the childhood. Related to this is the importance of physical education in schools and the promotion of participation in sport activities at school (Loprinzi et al., 2017).

According to the World Health Organization, the recommended physical activity for children and adolescents should correspond with 60 min of moderate-to-vigorous physical activity (MVPA) per day (WHO, 2010). This is equivalent of 12 000 steps a day and more. This result has been verified in practice in 6–19 years old children and adolescents (Colley et al., 2012).

Most research measuring the physical activity of children and adolescents with intellectual disability agrees that children with intellectual disability have much lower than recommended daily activity levels. They also have lower physical activity than their healthy peers (Einarsson et al., 2015; Foley et al., 2008; Frey et al., 2008). Most of the studies analyzed involved physical activity of children and adolescents with mild to moderate intellectual disability. There is a lack of data for children with severe intellectual disability (Leung et al., 2017).

Pitteti et al. (1989) was the first to measure physical activity of Special Olympics athletes with intellectual disabilities using an actigraph and compared them with people with intellectual disabilities who did not regularly practice sports.

In the United Kingdom, only 23 % of adolescents with intellectual disability achieved the recommended daily activity of 60 min of MVPA (Boddy et al., 2015). Icelandic children with intellectual disability have 40 % less physical activity than their peers and spend 9% more time per day being sedentary. In children with intellectual disability, no difference in physical activity was found between genders or between the weekday and the weekend. None of the children with intellectual disability met the recommended daily physical activity of 60 min of moderate to vigorous PA. While 40% of their non-disabled peers meets this standard (Einarsson et al., 2015). In Australia, only 42.1% of children and adolescents with mild to moderate intellectual disability met 60 min of MVPA each day (Shields et al., 2009). Only 47% of Dutch children and adolescents (aged 2–18) with moderate to severe intellectual disability fulfilled the recommended physical activity daily (Wouters et al., 2018).

Most of the data collected in the world are one-offs, experimental and cross-sectional, descriptive measurements. A minimum of authors presents research of physical activity in children and adolescents with mental disabilities using a longer-term perspective. In the Czech Republic, two-year follow-up of adults with mental disabilities was performed by Válková and Thaiszová (1989), who researched trends in Fitness indicators. The presented article shows the trends in weekly physical activity, so it is an innovation of long-term monitoring using movbands. It also shows the effect of school attendance (TV in the school curriculum) and holidays (leisure activities in the context of the family).

Other studies have shown the importance of gender in the level of physical activity. In the United States, boys with intellectual disability have been found to be more active than girls (Foley et al., 2008), as has been demonstrated in Spain (Izquierdo-Gomez et al., 2014, 2017). The reason for the gender difference may be more developed motor skills in boys than in girls with intellectual disability (Rintala and Loovis, 2013; Westendorp et al., 2014).

The level of physical activity in children and adolescents with intellectual disability decreases with age (Phillips and Holland 2011). This trend continues into adulthood, when an adult with intellectual disability engages in little or no physical activity (Finlayson et al., 2009; Hilgenkamp et al., 2012). Compared to their peers, children with intellectual disability are less active and participate in lower intensity physical activities (Stanish and Mozzochi 2000; Foley and McCubbin, 2009).

No research that measures physical activity of children with intellectual disability has been published in the Czech Republic. Only Válková, Králíková & Dygrýn (2018) measured-weekly with accelerometers - physical activity of people aged 14–35 with intellectual disability at a sport camp and again with the same participants in the home environment. Physical activity in men (average 5 000 steps per day) and in women (average 4 000 steps per day) was again half the norm. As in the general population, men had higher physical activity than women by about 1 000 steps a day. Válková, Qu & Chmelík (2014) found that the differences between men and women were statistically insignificant in terms of the recommended daily physical activity (10 000 steps) in adult athletes of Special Olympians during SO competitions.

None of the published studies deal with the longitudinal investigation of the same participants with intellectual disability. Also, none of the published research shows trends in physical activity, BMI and muscles in children and adolescents. Comparable data is completely missing in this area.

The first aim of the research was to identify the trends in physical activity, BMI and muscles in children and adolescents with ID in the Czech Republic over 2-year period and find out whether two month summer holidays have effect on development trends. The second aim was to find out the level of physical activity of children and its correlation with BMI and muscles. The third aim was to find out a correlation between postural balance and children's muscles.

## 2 Methods

The research was longitudinal, it took place at two primary schools in the Czech Republic in the Zlín Region over two years. Based on an agreement with ČHSO (Czech Special Olympics Movement), the research was part of the Czech Healthy Community Project (Y2-17-600-11) in 2016–2019 and was funded by the US Golisano Foundation. This is a completely new study on indicators of fitness and physical activity.

The data was collected a total of four times across the two years, each year in June and September, to find out whether the summer holidays influenced the physical activity of the pupils. *Note: in the Czech Republic, pupils have* 2 months *of summer holidays (July, August), during which pupils are at home and do not go to school. The school year runs from September to June.*

**Figure 1:**
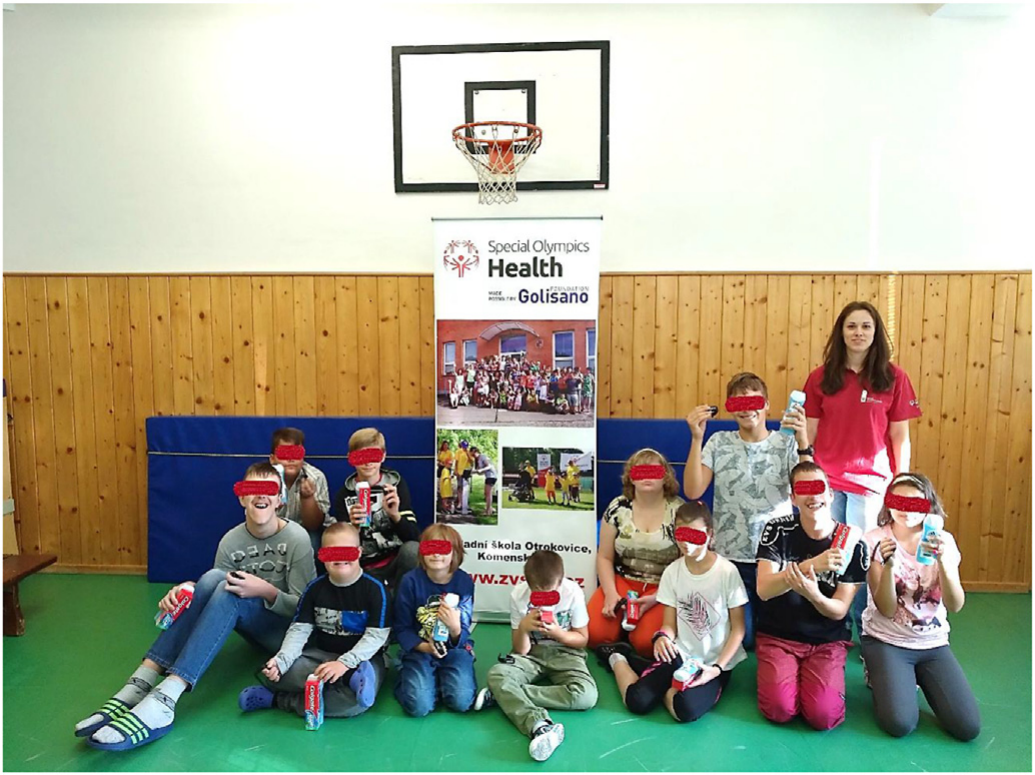
The reward for wearing movbands. (Note: Parental consent is part of the school's documentation for given school year 2017/2018 and 2018/2019 and is stored at the school).

Thanks to the project funding, it was possible to motivate research participants for their participation with a reward. Pupils who wore the movband all week received a complimentary toiletries package which included, for example, a shower gel and a toothpaste (Figure 1). This was a great motivation for the pupils and the participation in the research became a prestigious matter for them.

### 2.1 Participants

The participants are pupils from two primary schools in the Zlín Region in the Czech Republic. Most pupils have a simple intellectual disability, i.e. without other associated conditions (such as epilepsy, Down syndrome, autism). No child in the research had Down Syndrome. Pupils are aged 8–19 (Table 1) and have a mild and moderate degree of intellectual disability. Intellectual disability is congenital in all children.

**Table 1:** Characteristics of participants by age.

Category/Measurement	June 2017	September 2017	June 2018	September 2018
	M ± SD	M ± SD	M ± SD	M ± SD
Girls	13.25 ± 2.90	14.18 ± 2.67	15.07 ± 2.71	14.4 ± 2.84
Boys	12.00 ± 2.28	12.60 ± 2.50	12.95 ± 2.75	13.00 ± 2.43
Moderate ID	11.50 ± 2.68	13.79 ± 2.86	13.95 ± 3.19	14.50 ± 2.66
Mild ID	13.50 ± 1.58	12.43 ± 2.16	13.14 ± 2.18	12.07 ± 1.98

Note: M = median, SD = standard deviation.

Children with moderate ID are educated at school together with children with mild ID according to the school educational program, which is regulated in the Education Law.

The consent of the Faculty of Sports Studies (FSpS) ethics committee was given for the research, the consent of the headteachers of the participating schools was given and the parents of the pupils signed consent forms.

The following table (Table 2) shows the examined group according to the number of participants in each category. Around 30 children with intellectual disability participated in each data collection period. (7 pupils were excluded from the study in June 2017, 4 pupils in September 2017, 1 pupil in June 2018 and 3 pupils in September 2018) because they did not wear movbands. These pupils are not included in the table. There were also some research participants missing from school during each of the data collection periods.

**Table 2:** Characteristics of participants according to the number of participants.

Category/Measurements	June 2017	September 2017	June 2018	September 2018
Girls	12	17	15	11
Boys	11	20	21	22
**Total girls and boys**	**23**	**37**	**36**	**33**
Moderate ID	13	21	22	19
Mild ID	10	16	14	14
**Total pupils of moderate and mild ID**	**23**	**37**	**36**	**33**

Summary of the number of participants in the research according to the levels of mental disability.

### 2.2 The process of data collection and the assessment of physical activity

**Figure 2:**
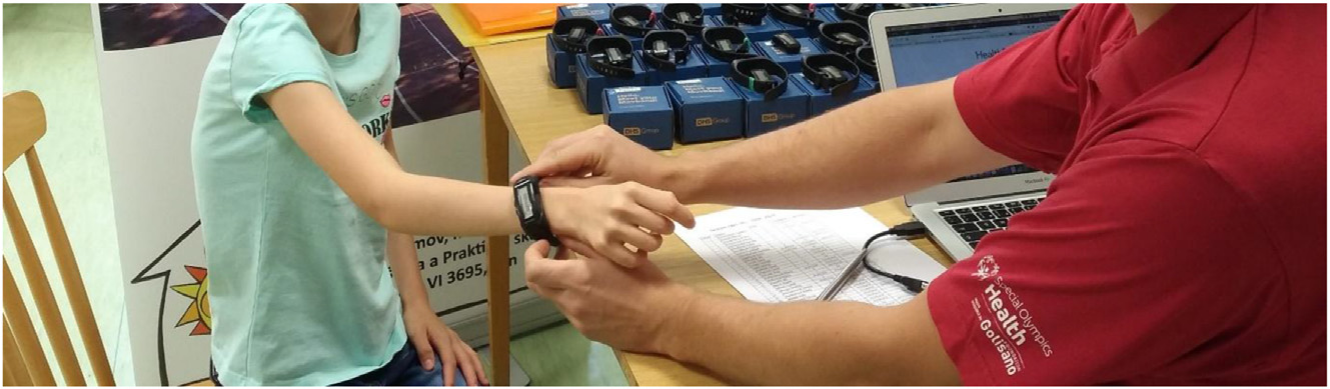
Movband setup and synchronization. (Note: Parental consent is part of the school's documentation for given school year 2017/2018 and 2018/2019 and is stored at the school).

Weekly physical activity level was determined by wearing a so-called movband watch. Movband (model Movband 2) represents the first convergence of the latest accelerometer technology and a stylish and comfortable wear. The advantage of movband is its simplicity, accuracy, style and affordability. Movband is suitable for children of all ages and is an easy way to monitor their physical activity (Motion Fitness, 2010). The Movband is placed on the user's wrist like a watch (Figure 2). It is much more comfortable for the user than a fixed accelerometer worn behind the waist. Based on the height and gender, the movband calculates the stride length.

Movband records all movement, i.e. it evaluates the number of steps, kilometers travelled and hand movements. It also shows the time. The children were informed that they had to wear the movband on their wrist for one week. Every day they put on the movband in the morning after waking up and took it off in the evening before showering (the movband is not waterproof). We created written instructions for the children and parents.

The use of accelerometers has been validated in both children and adolescents with physical disabilities (Clanchy et al., 2011) and with intellectual disability (McGarty et al., 2016). Movband (model Movband 2) provides a valid rating for measuring physical activity. There is a strong and significant correlation between actigraph and movband (r = 0.97, p < 0.001), (Barkley et al., 2018).

### 2.3 Measurement of BMI and muscle mass

**Figure 3:**
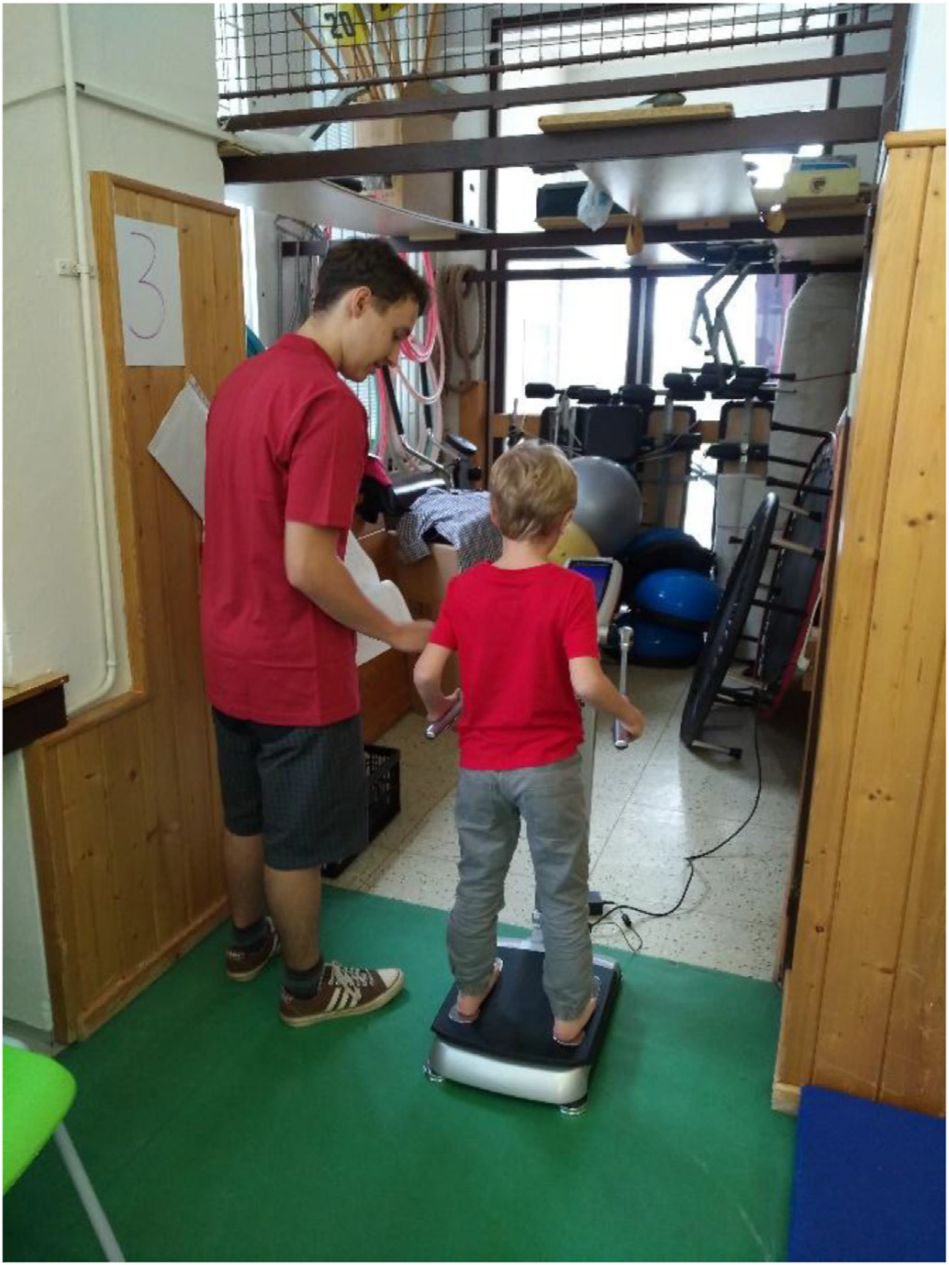
BMI scanning with InBody 230 analyser.

The InBody 230 analyser was used to determine the exact body composition – BMI index and muscle mass. InBody uses the method of 8-point touch electrodes, thanks to which it measures the body in segments. In the picture (Figure 3) we see an example of measurements at a special needs primary school. The measurements took place in the gym of the school and were spread over two days each time, one day in each school.

BMI refers to body mass index and is used to indicate underweight, normal weight and obesity. There is a universal table for the adult population that does not consider age or gender. There is no universal table for children aged 5 to 19. According to the measured BMI value, one of the categories is then determined from the graph according to gender and age: obesity, normal weight, overweight, underweight and significant underweight in which the child is included. The graphs were compiled by the WHO (World Health Organization) and are for girls and boys separately. BMI shows the value in kg/m^2^ and can also be calculated using the formula: $BMI=\frac{weight\;in\;kg}{{\left(height\;in\;metres\right)}^{2}}$, (WHO, 2018).

The muscle mass of the InBody is measured directly in kg and no conversion is required.

### 2.4 Postural balance test

**Figure 4:**
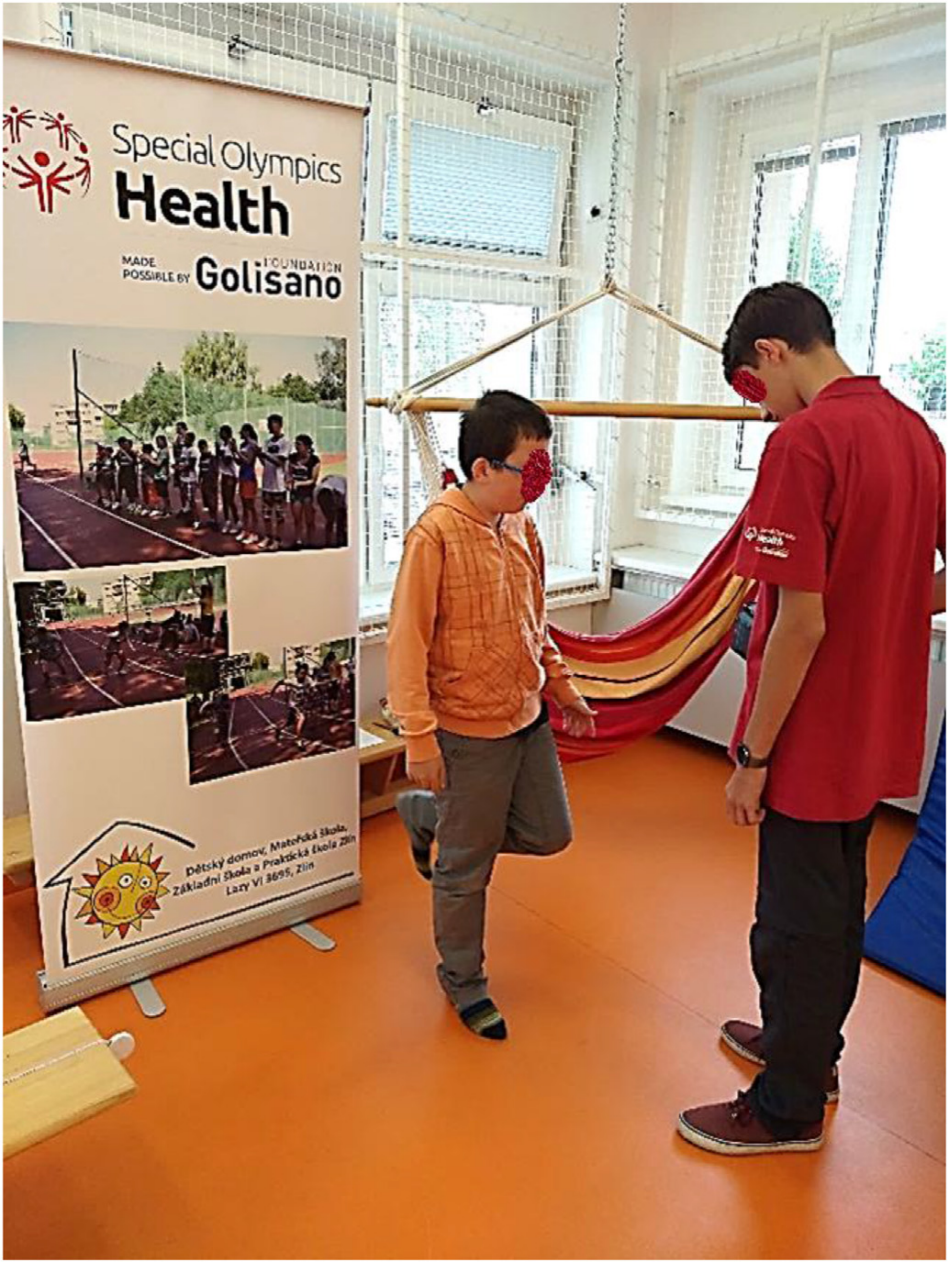
Single leg stance with eyes open.

It is called a Single leg stance with eyes open. It is a simple method of quantifying balance using visual stimuli. In the postural balance test a person stands on 1 leg with their eyes open (Figure 4). The hands are sideways or alongside the body. They fix their gaze on a marked point on the wall. During the test, a coach encourages the athlete and is ready to give them help.

We measure the time before the balance is lost or until the person touches the ground with both feet. The goal is to stay in this position as long as possible, the maximum is 30 s, then the test ends.

This test is standardized and used as standard in FUNfitness Special Olympics tests worldwide (Special Olympics FUNfitness, 2013, p. 48).

### 2.5 Data processing and analysis

The children wore movbands for one week. The total volume of physical activity was processed as the median number of steps per day, which was determined from the weekly physical activity data. All 3 available tests in the program Statistica 12 were used to determine the normality of the data: Kolmogorov-Smirnov, Lilliefors and Shapiro-Wilks Test. Based on all 3 tests, it was found that these are non-parametric data. (I.e. the data do not correspond to the Gaussian curve of the normal distribution). Therefore, we use the median in the graphs, which is a nonparametric quantity.

Furthermore, the analysis of discrepancies was performed (because there is a total of 4` measurements at each school), specifically Friedman anova, which shows a statistically significant difference at the 5% level of significance, i.e. for the values of p ≤ 0,05. A Wilcoxon's t-test was also performed, for non-parametric data, to find out which measurements have statistically significant differences between the two years measured.

To determine the connection between the observed quantities, a correlation was used, namely the Spearman correlation coefficient, which is also used for nonparametric data.

Each research area was evaluated in terms of developing trends in the whole of the research period. Based on the Švancara (1980, p. 20) and Válková and Thaiszová (1989) categorization of the development trends, 6 basic trends were analyzed: positive, negative, unbalanced, stable, concave, convex.

Trends were also processed in the program Statistica 12. They are shown in the form of box graphs, where it is possible to see the minimum and maximum value in the file. To represent the center of the trend, the median is always used, which is a nonparametric quantity and is not distorted by extreme values. The trend is a curve that connects the median values for the individual measurements in the graphs.

## 3 Results

The results will be presented in the following sequence:

3.1 Trends in physical activity, BMI and muscles by sex
3.2 Trends in physical activity, BMI and muscles according to the degree of intellectual disability
3.3 Analysis of Friedman anova and T-test by sex and by the degree of intellectual disability
3.4 Comparison and correlation of postural balance with muscles

### 3.1 Trends in physical activity, BMI and muscles by sex

#### 3.1.1 The trends in girls

**Figure 5:**
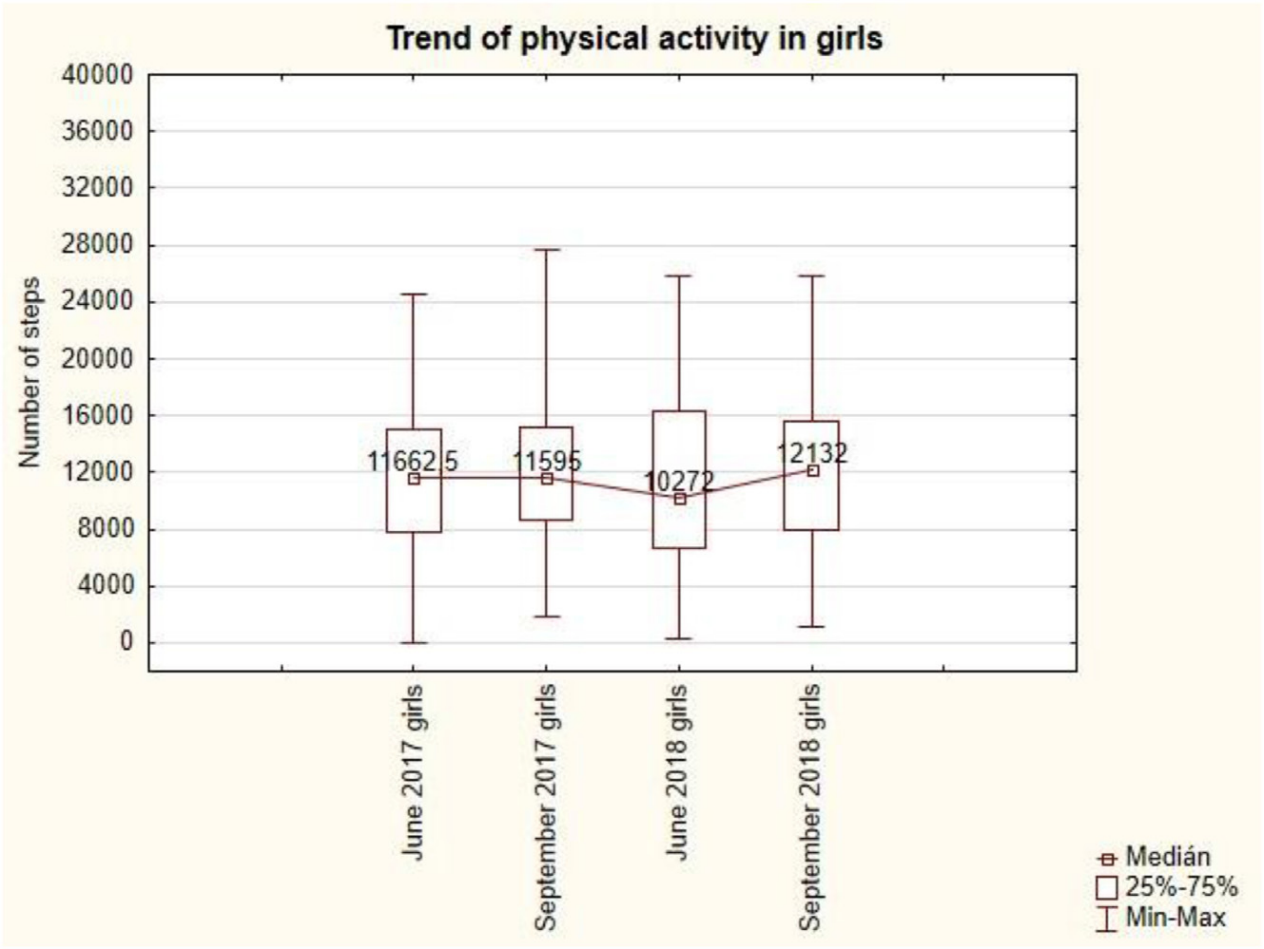
The trend of weekly physical activity in girls. Note: A trend is a curve that can have different shapes.

The trend of physical activity in girls (Figure 5) is convex. The lowest value of 10 272 steps is in June 2018 and the highest value of 12 132 steps is in the last measurement in September 2018. The summer holidays in September 2017 are followed by a decrease in the number of steps to 11 595 per day, but the summer holidays in September 2018 are followed by an increase in the number of steps to 12 132 per day. Summer holidays do not have a demonstrable effect on the mean value of the number of steps per day during the week.

**Figure 6:**
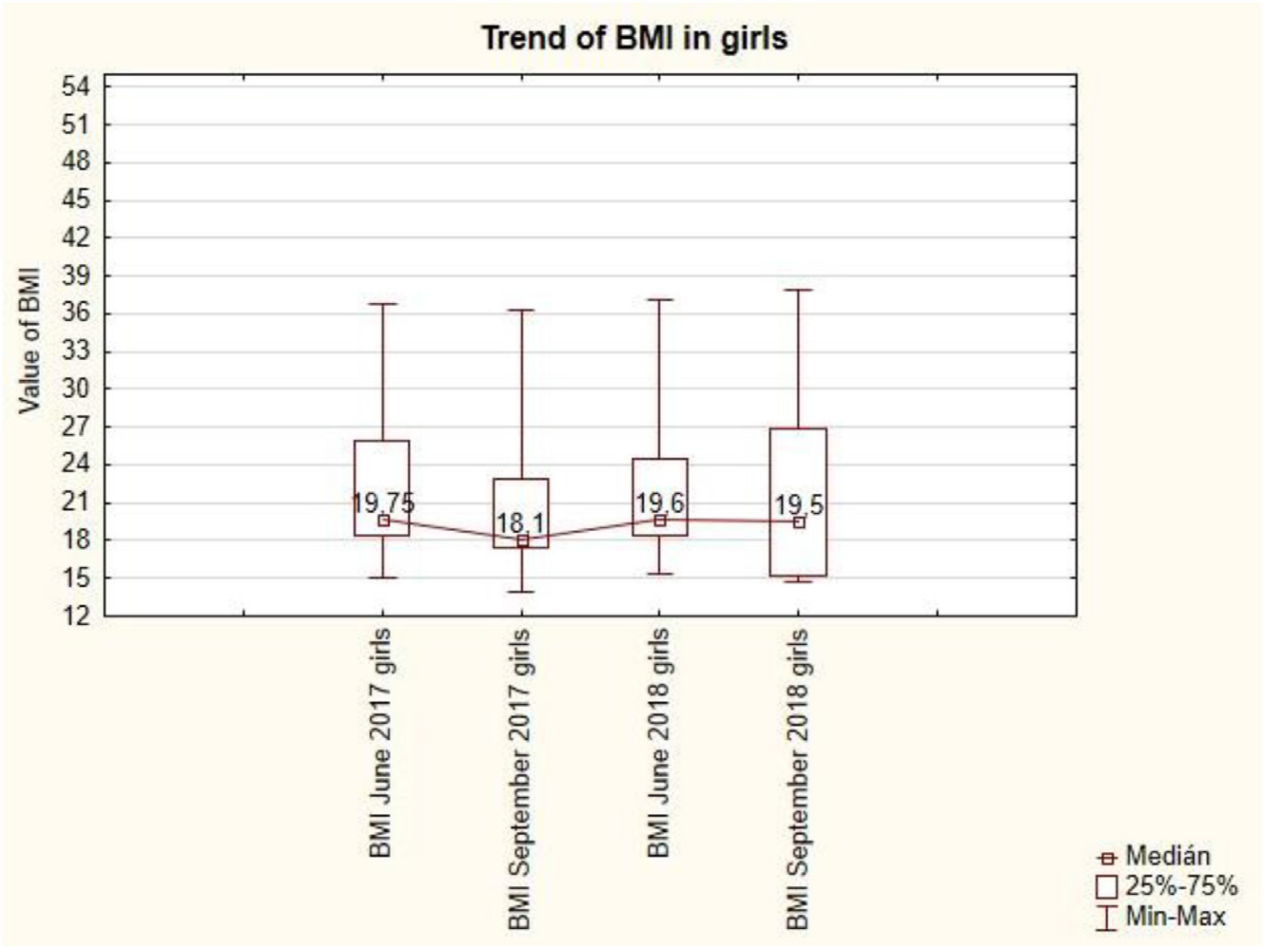
The trend of BMI in girls.

The trend of BMI in girls is unbalanced (Figure 6). After the summer holidays in September, BMI always decreases (from 19.75 to 18.1 and from 19.6 to 19.5). Summer holidays cause a decrease in BMI values in girls. This change is not statistically significant (see Table 7).

**Figure 7:**
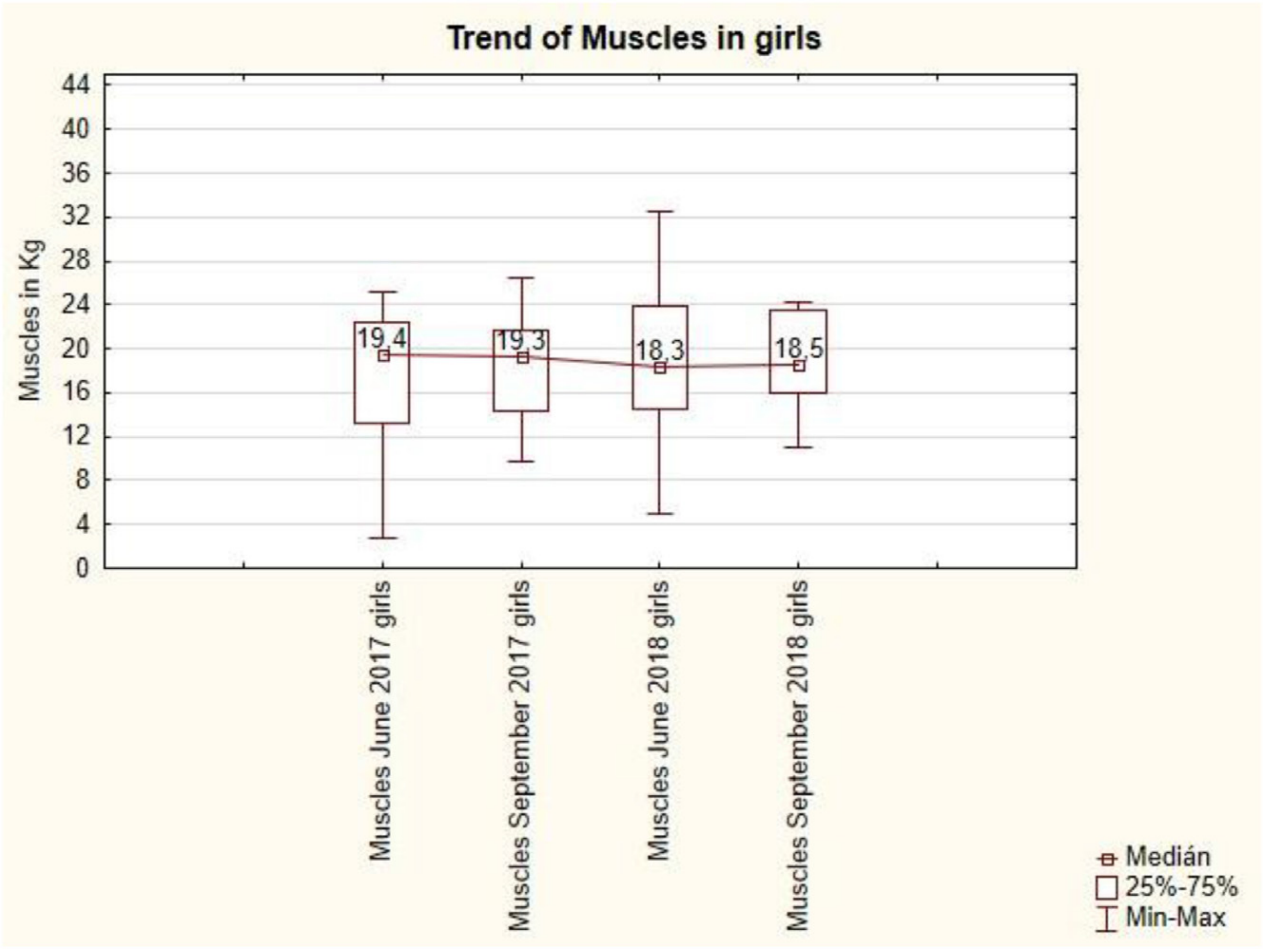
The trend of muscles in girls.

The trend of muscles in girls is convex (Figure 7). After the summer holidays in September 2017, there is a slight loss of muscle from 19.4 kg to 19.3 kg, but in September 2018 there is an increase in muscle from 18.3 to 18.5 kg. Summer holidays do not have a demonstrable effect on the value of muscles in girls.

#### 3.1.2 The trends in boys

**Figure 8:**
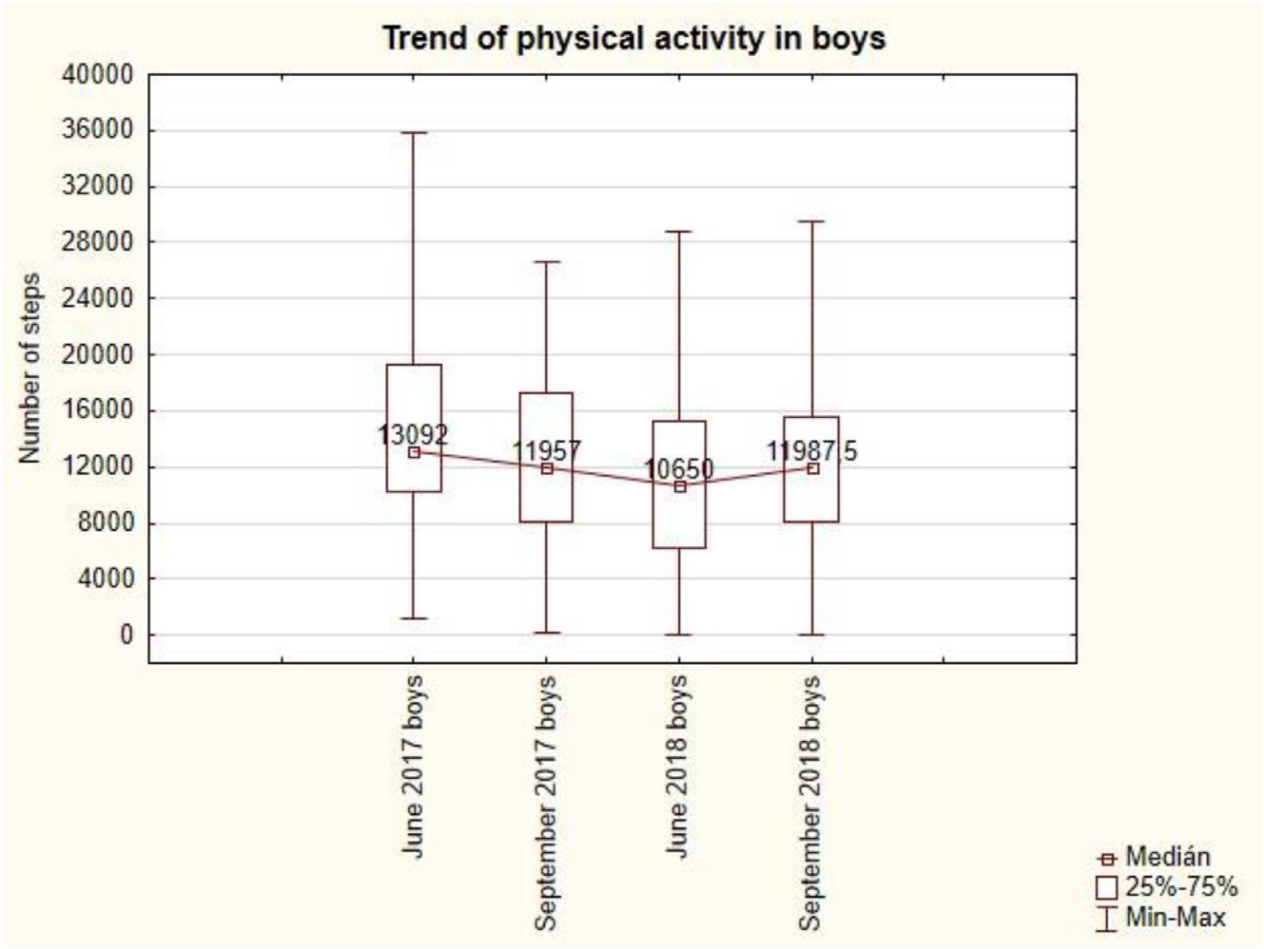
The trend of weekly physical activity in boys.

The trend of physical activity in boys is convex (Figure 8). The lowest value of 10 650 steps per day is in June 2018 and the highest value of 13 092 steps is in the first measurement in June 2017. The summer holidays in September 2017 are followed by a decrease in the number of steps to 11 957 per day, but the summer holidays in September 2018 are followed by an increase in the number of steps to 11 987.5 per day. Summer holidays do not have a demonstrable effect on the mean value of the number of steps per day during the week.

**Figure 9:**
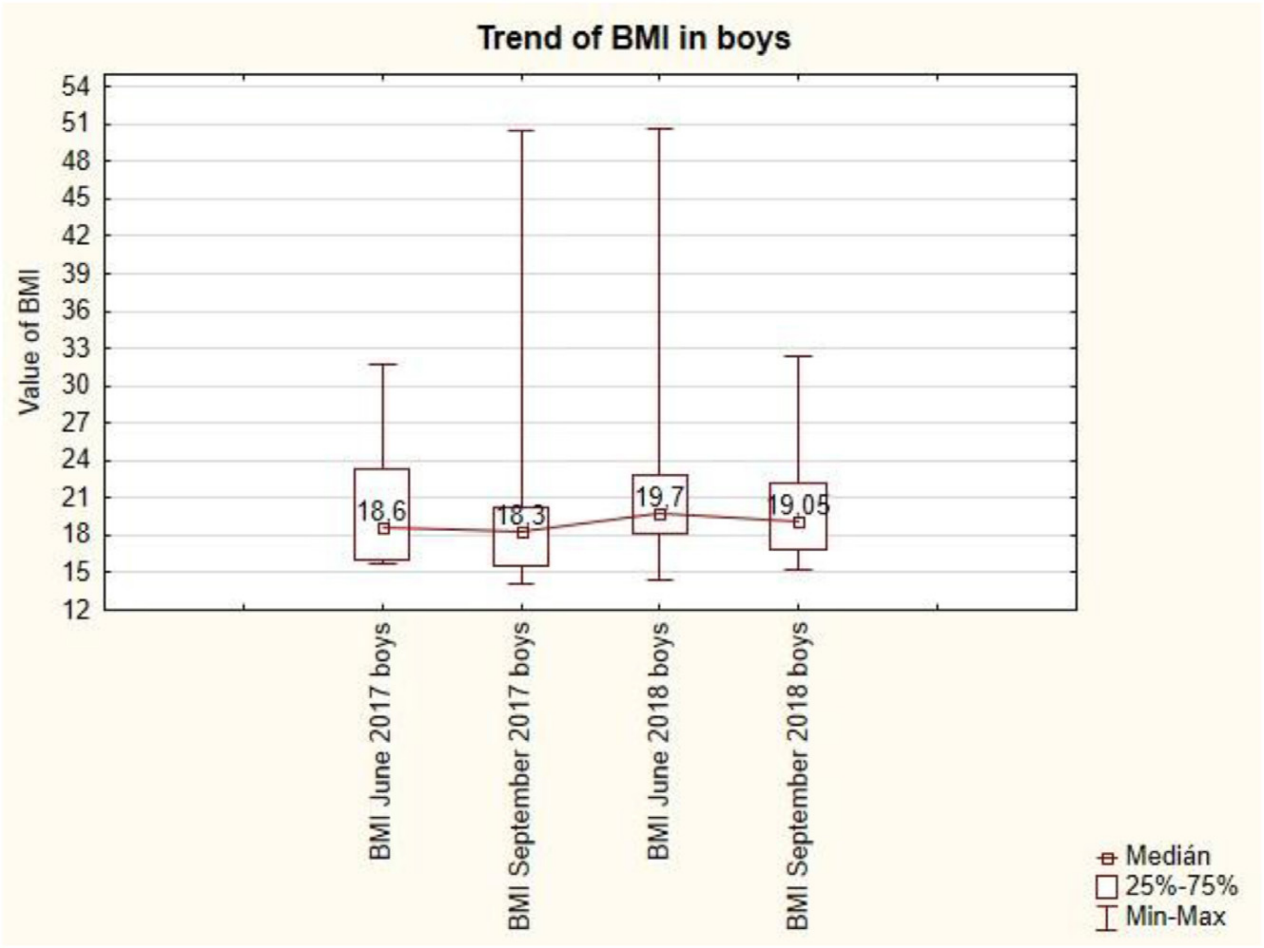
The trend of BMI in boys.

The trend of BMI in boys is unbalanced (Figure 9). After the summer holidays in September, BMI always decreases (from 18.6 to 18.3 and from 19.7 to 19.05). Summer holidays cause a decrease in BMI values for boys. This change is not statistically significant (see Table 7).

**Figure 10:**
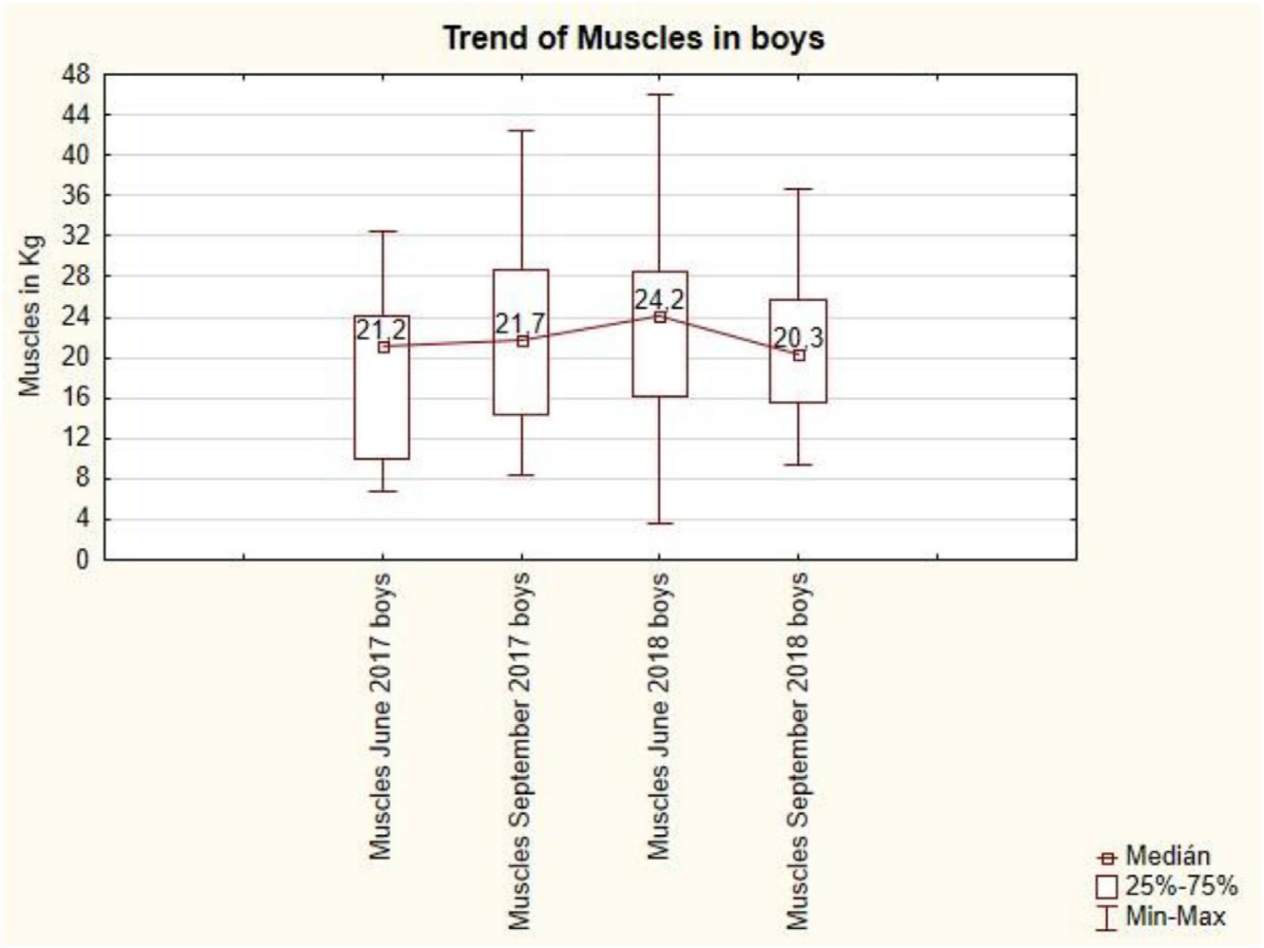
The trend of muscles in boys is.

The trend of muscles in boys is concave (Figure 10). After the summer holidays in September 2017, the muscles increased from 21.2 kg to 19.7 kg, but in September 2018, the muscles decreased from 24.2 kg to 20.3 kg. Summer holidays do not have a demonstrable effect on muscle value in boys.

#### 3.1.3 A comparison of trends by sex with the norm and correlation

For children, the recommended standard is 12 000 steps per day. The mean value is always considered to be the median with respect to the nonparametric data.

In the following table (Table 3) we can see that girls exceeded the recommended daily norm only once and it was only by 1%. In other cases, they reached 86 % and 97 % of the recommended daily activity. The boys had physical activity within the norm in September. Only in one measurement they were below the norm and reached only 89 % of the recommended physical activity. Once they reached 9 % above the norm.

**Table 3:** A comparison of PA and BMI by sex with the norm.

Physical activity - Steps	SD - Girls	Median-Girls	Girls in % of norm	SD - Boys	Median - Boys	Boys in % of norm
June 2017	±5 159,05	11 662,50	**97 %**	±7 174,50	13 092	**109 %**
September 2017	±5 371,18	11 595	**97 %**	±5 771,44	11 957	**100 %**
June 2018	±5 964,82	10 272	**86 %**	±6 495,40	10 650	**89 %**
September 2018	±6 177,15	12 132	**101 %**	±5 793,83	11 987,50	**100 %**
BMI	SD - Girls	Median -BMI Girls	Category - Girls	SD - Boys	Median -BMI Boys	Boys in % of norm
June 2017	±6,29	19,75	**normal weight**	±5,75	18,60	**normal weight**
September 2017	±6,18	18,10	**normal weight**	±8,00	18,30	**normal weight**
June 2018	±5,50	19,60	**normal weight**	±7,92	19,70	**normal weight**
September 2018	±6,94	19,50	**normal weight**	±4,30	19,05	**normal weight**

SD = standard deviation.

Highlighting the norm of physical activity and BMI category in girls and boys.

In terms of BMI values, both girls and boys were in the normal weight category throughout the measurement. BMI values are similar in both sexes.

The correlation (Table 4) between the physical activity of girls and boys and their BMI ranges from a moderately negative correlation (r = -0.411 for girls in June 2018, thus, the more physical activities they have, the lower their BMI). The correlation ranges up to a positive weak correlation (r = 0.300 boys in September 2018, thus, the more physical activities they have, the higher their BMI).

**Table 4:** Correlation of physical activity with BMI and muscles by sex.

Spearman correlation coefficient	Physical activity			
p < 0,05	June 2017	September 2017	June 2018	September 2018
BMI girls	-0,235	0,147	-0,411	-0,370
BMI boys	0,073	-0,063	-0,332	0,300
Muscles girls	-0,154	-0,093	-0,173	0,055
Muscles boys	0,009	0,021	**-0,517**	-0,204

I highlighted the stronger value of the correlation.

The correlation between the physical activity of girls and boys and their muscles ranges from the absence of a correlation (r = 0.009) to a moderately negative correlation (r = -0.516 for boys in June 2018).

### 3.2 Trends in physical activity according to the degree of intellectual disability

#### 3.2.1 The trends in pupils with mild ID

**Figure 11:**
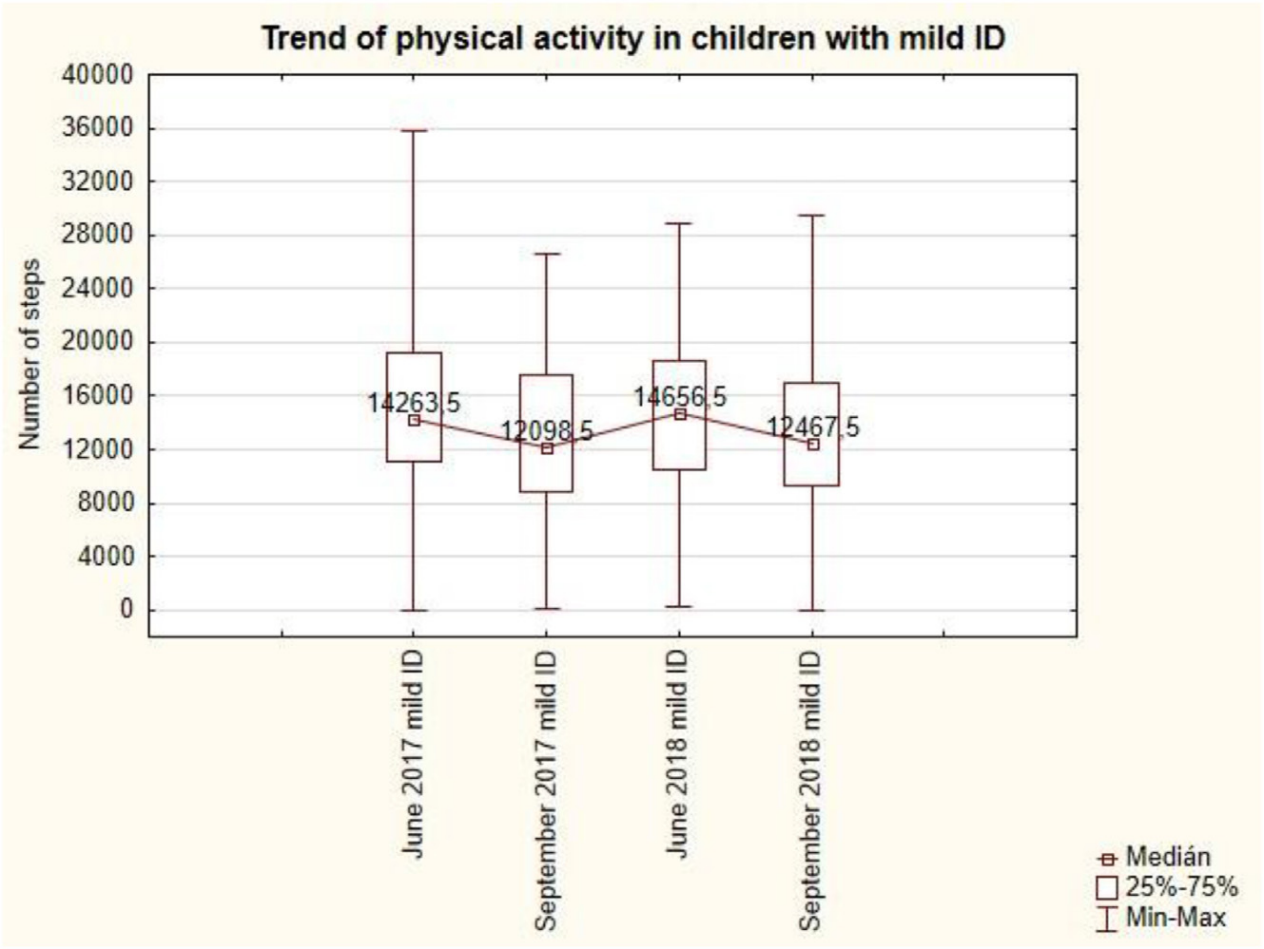
The trend of physical activity in pupils with mild intellectual disability.

The trend of PA for mild disability (Figure 11) is uneven. The lowest value of 10 098.5 steps per day is in September 2017 and the highest value is 14 656.5 steps per day in June 2018.

The summer holidays in September 2017 are followed by a significant decrease in the number of steps to 12 098.5 and also the summer holidays in September 2018 are followed by a decrease in the number of steps to 12 467.5. Summer holidays have a demonstrable effect – a reduction - on the daily number of steps during the week in the mild intellectual disability. This change is not statistically significant (see Table 7).

**Figure 12:**
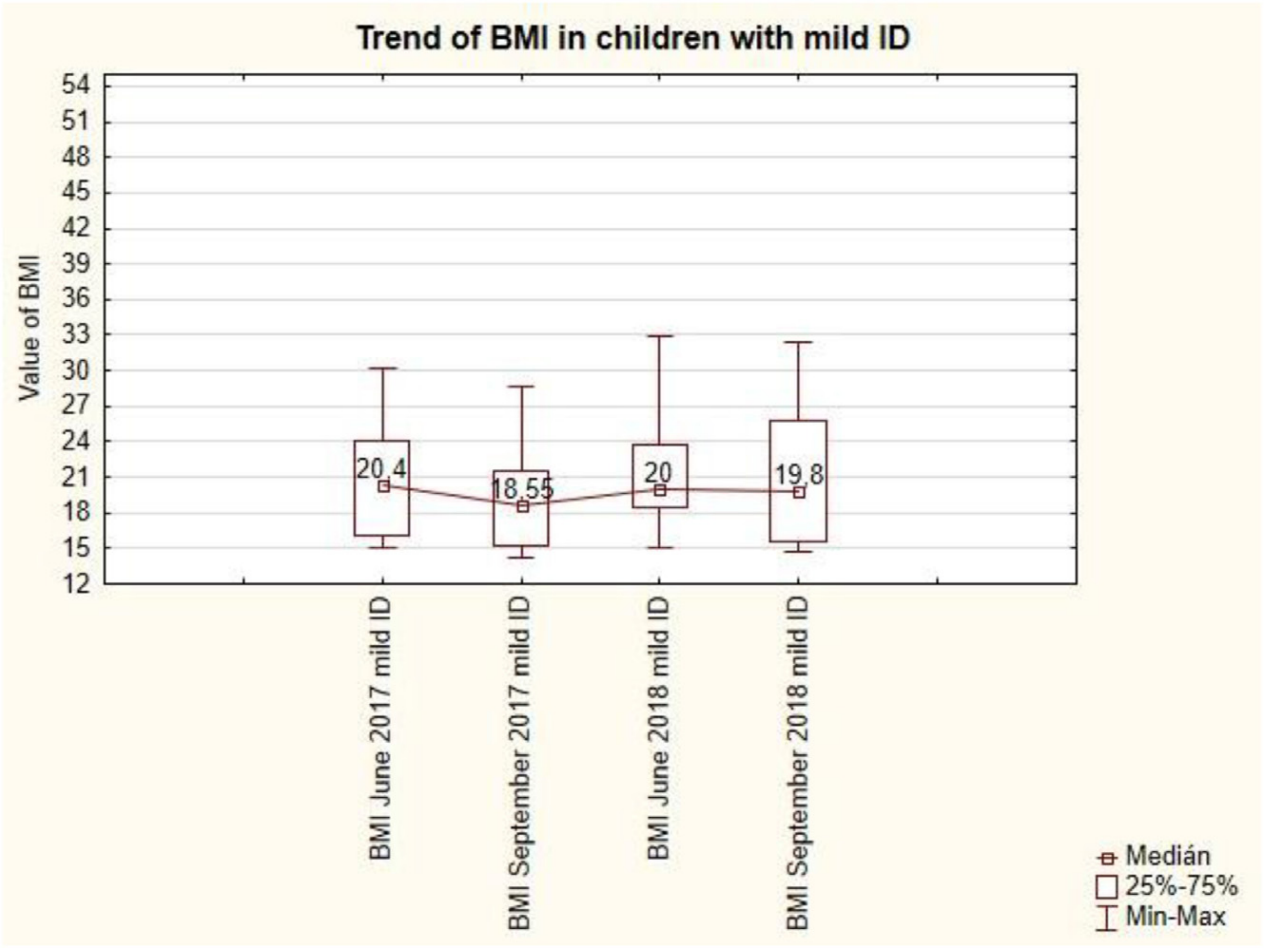
The trend of BMI in children with mild ID.

The trend of BMI in pupils with mild ID is uneven (Figure 12). After the summer holidays in September, BMI values always decrease (from 20.4 to 18.55 and from 20 to 19.8). Summer holidays cause a decrease in BMI values in pupils with mild ID. This change is not statistically significant (see Table 7).

**Figure 13:**
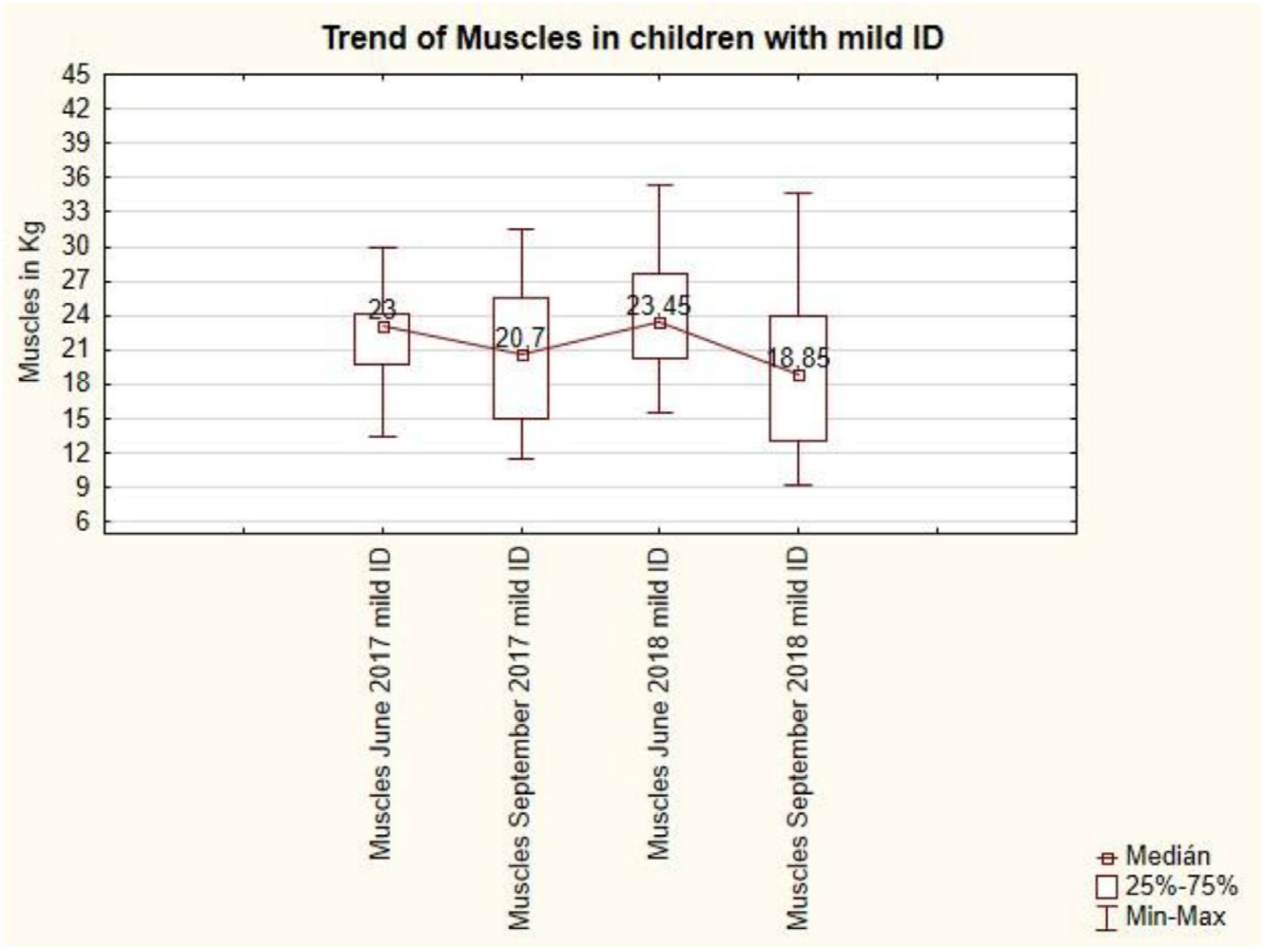
The trend of muscles in children with mild ID.

The muscle trend in pupils with mild ID is uneven (Figure 13). After the summer holidays in September 2017, there is a loss of muscle from 23 kg to 20.7 kg, also after the summer holidays in September 2018, there is a loss of muscle from 23.45 kg to 18.85 kg. Summer holidays cause muscles loss in pupils with mild ID. This change is not statistically significant (see Table 7).

#### 3.2.2 The trends in pupils with moderate ID

**Figure 14:**
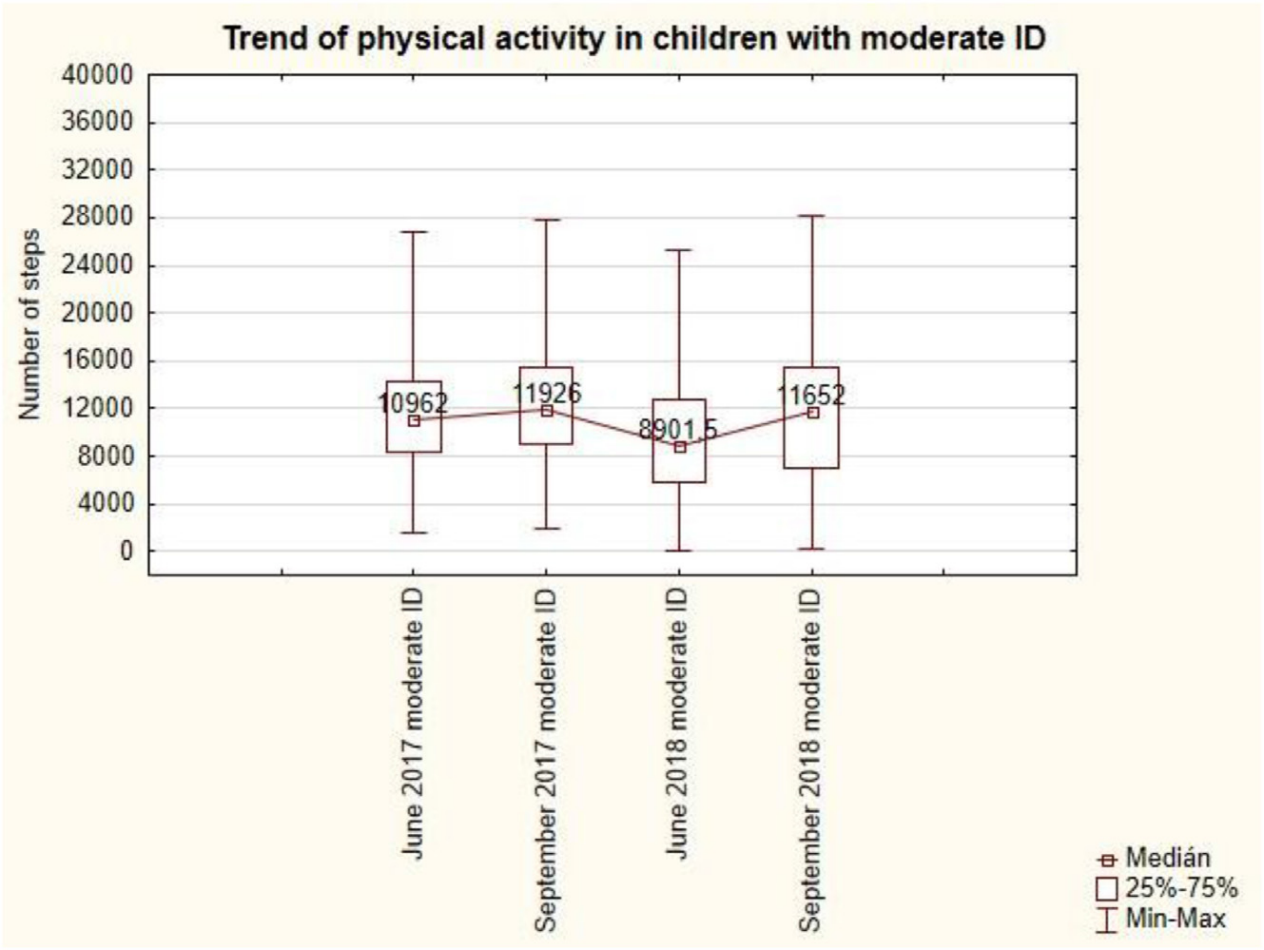
The trend of physical activity in pupils with moderate ID.

The trend of PA for moderate intellectual disability is also unbalanced (Figure 14). The lowest value is 8 901.5 steps per day is in June 2018 and the highest value is 11 926 steps per day in September 2017.

The summer holidays in September 2017 are followed by a slight increase in the number of steps to 11 926 per day, and the summer holidays in September 2018 are also followed by an increase in the number of steps to 11 652 per day. Summer holidays have a demonstrable effect – an increase on the daily number of steps during the week in the moderate intellectual disability. This change is statistically significant (see Table 7).

**Figure 15:**
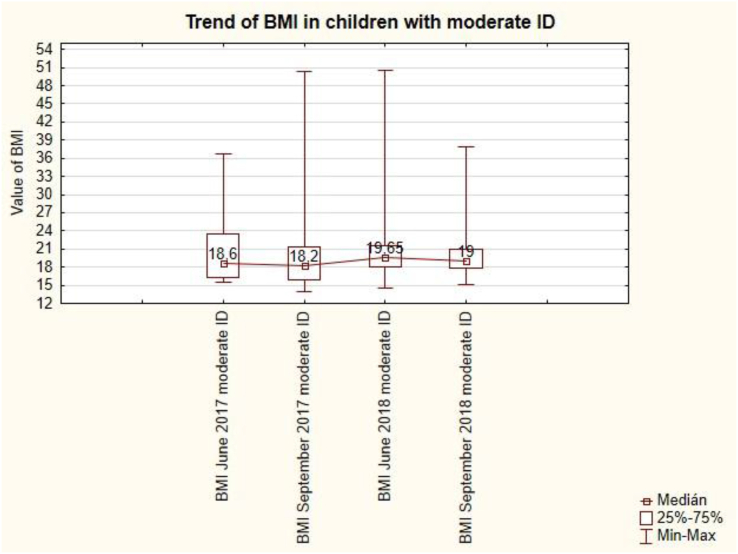
The trend of BMI in children with moderate ID.

The trend in pupils with moderate ID is uneven (Figure 15). After the summer holidays in September, BMI values always decrease (from 18.6 to 18.2 and from 19.65 to 19). Summer holidays cause a decrease in BMI values for pupils with moderate ID. This change is not statistically significant (see Table 7).

**Figure 16:**
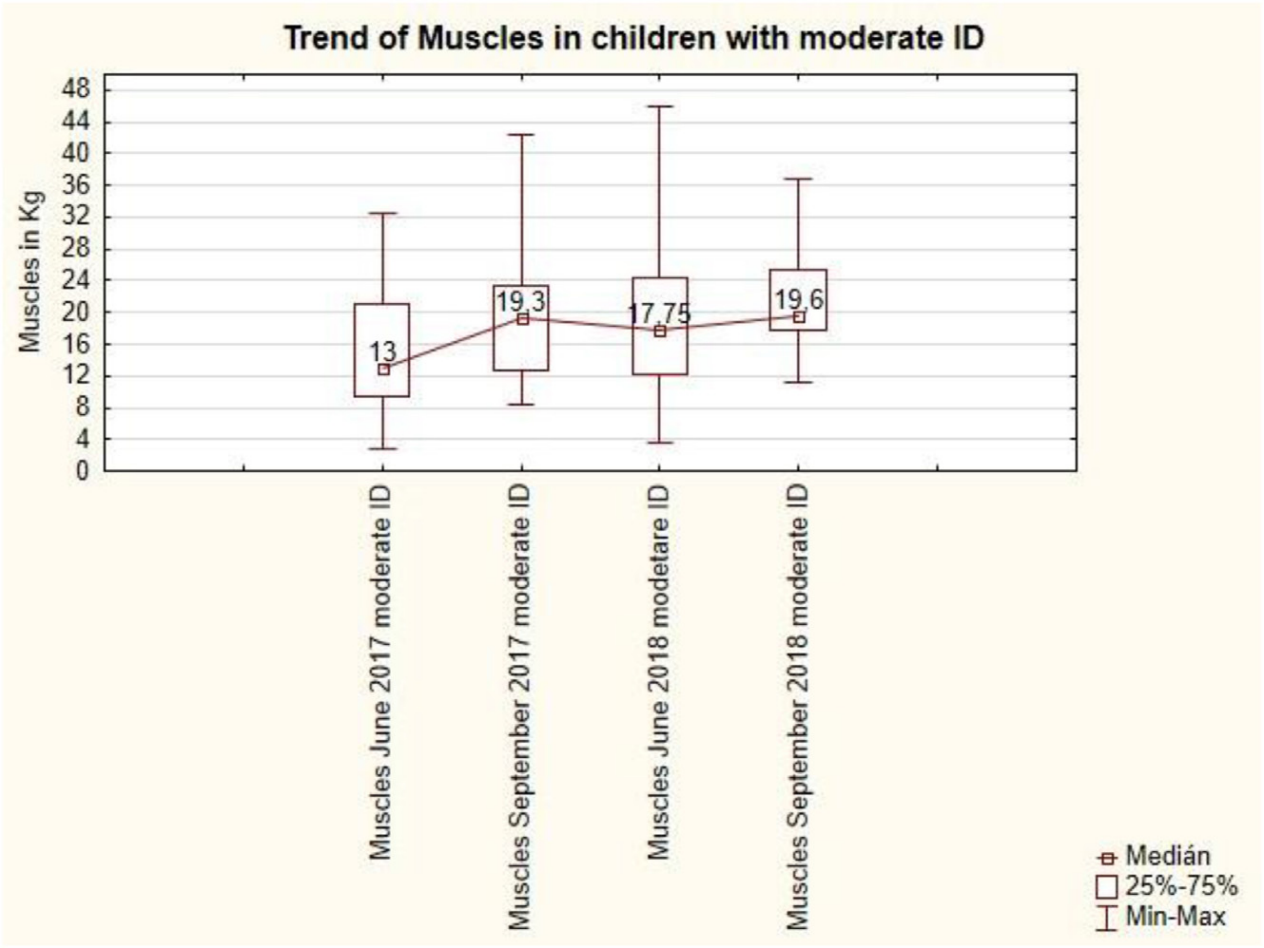
The trend of muscles in children with moderate ID.

The trend of muscles in pupils with moderate ID is unbalanced (Figure 16). After the summer holidays in September 2017, the muscles increased from 13 kg to 19.3 kg, also after the summer holidays in September 2018, the muscles increased from 17.75 kg to 19.6 kg. Summer holidays cause muscle growth in students with moderate ID. This change is statistically significant (see Table 7).

#### 3.2.3 A comparison of trends according to the degree of disability with the norm and correlation

We can see that pupils with a mild intellectual disability have particularly good physical activity levels, and in all measurements their daily number of steps exceeded the norm and reached 101 %–122 % of the norm (Table 5). We can also notice that they always had increased physical activity in June rather than in September. Pupils with moderate intellectual disability did not exceed the norm in any of the measurements, they reached 74 %–99 % of the recommended daily norm. However, these pupils always had increased physical activity in September.

**Table 5:** A comparison of PA and BMI according to the degree of disability with the norm.

Physical activity - Steps	SD - Mild ID	Median - Mild ID	Mild ID in % of norm	SD - Moderate ID	Median - Moderate ID	Moderate ID in % of norm
June 2017	±7 122,91	14 263,50	**119 %**	±4 992,33	10 962	**91 %**
September 2017	±6 062,35	12 098,50	**101 %**	±5 045,24	11 926	**99 %**
June 2018	±6 309,38	14 656,50	**122 %**	±5 408,89	8 901,50	**74 %**
September 2018	±5 515,99	12 467,50	**104 %**	±6 053,42	11 652	**97 %**
BMI	SD - Mild ID	Median - BMI Mild ID	Category - Mild ID	SD - Moderate ID	Median - BMI Moderate ID	Category - Moderate ID
June 2017	±5,08	20,40	**normal weight**	±6,73	18,60	**normal weight**
September 2017	±4,27	18,55	**normal weight**	±8,75	18,20	**normal weight**
June 2018	±4,94	20	**normal weight**	±8,05	19,65	**normal weight**
September 2018	±5,54	19,80	**normal weight**	±5,16	19	**normal weight**

SD = standard deviation.

Highlighting the norm of physical activity and BMI category in mild ID and moderate ID.

In terms of BMI values, pupils with mild and moderate ID were in the normal weight category throughout the measurement. Pupils with moderate ID have slightly lower BMI values in each measurement than pupils with mild ID. Pupils' BMI values are again remarkably similar.

The correlation (Table 6) between physical activity in the mild and moderate ID and their BMI ranges from a positive mean correlation (r = 0.371 moderate ID in September 2018) to a mean negative correlation (r = -0.399 moderate ID in June 2018).

**Table 6:** Correlation of PA with BMI and muscles according to the degree of disability.

Spearman correlation coefficient	Physical activity			
p < 0,05	June 2017	September 2017	June 2018	September 2018
BMI mild ID	0,152	0,012	-0,181	0,130
BMI moderate ID	-0,085	-0,120	-0,399	0,372
Muscles mild ID	0,103	0,326	**-0,596**	-0,249
Muscles moderate ID	-0,126	-0,131	-0,302	0,068

I highlighted the stronger value of the correlation.

The correlation between physical activity in the mild and moderate ID and their muscles also ranges from a medium positive correlation (r = 0.326 mild ID in September 2017) to a strong negative correlation (r = -0.595 mild ID in June 2018).

### 3.3 An analysis of the Friedman anova and the T-test by sex and by the degree of intellectual disability

The following table (Table 7) shows the results of the variance, specifically the Friedman anova, for all four measurements at each school. In terms of weekly physical activity (p = 0,00129) and muscles (p = 0,03255), there was a statistically significant difference of 5 % during the two-year data collection in pupils with moderate intellectual disability. There is no statistically significant difference for the other categories.

**Table 7:** Friedman's anova in girls, boys, mild ID and moderate ID.

Category/Value	p
Physical activity - Girls	0,65998
Physical activity - Boys	0,07324
Physical activity - Mild ID	0,23725

**Physical activity - Moderate ID**	**0,00129**
BMI - Girls	0,74378
BMI - Boys	0,31492
BMI - Mild ID	0,61494

BMI - Moderate ID	0,59506
R - leg - Girls	0,07575
L - leg - Girls	0,36011
R - leg Boys	0,66454
L - leg - Boys	0,92776
R - leg - Mild ID	0,18062
L - leg - Mild ID	0,50821
R - leg - Moderate ID	0,28425

L - leg - Moderate ID	0,91801
Muscles - Girls	0,96507
Muscles - Boys	0,87106
Muscles - Mild ID	0,25296
**Muscles - Moderate ID**	**0,03255**

Highlighting of statistically significant differences at the 5% level of significance.

The Wilcoxon T-test, which is also nonparametric and is used to compare dependent groups, was used to find statistically significant differences between each measurement in the two year period. In the table (Table 8) we can see the resulting values at 5 % significance. Statistically significant differences in the physical activity in pupils with moderate intellectual disability are all consecutive measurements. For the other categories of PA, there are no statistically significant differences between the measurements.

**Table 8:** The Wilcoxon T-test for each category.

Physical activity -T-test between measurements	Girls	Boys	Mild ID	Moderate ID
in each category	p	p	p	p
June 2017 + September 2017	0,079656	0,989872	0,913785	**0,008964**
September 2017 + June 2018	0,137566	0,149447	0,603668	**0,000373**
June 2018 + September 2018	0,888952	0,166774	0,067744	**0,015053**
BMI -T-test between measurements	Girls	Boys	Mild ID	Moderate ID
in each category	p	p	p	p
June 2017 + September 2017	0,084380	0,328066	0,798860	0,552494
September 2017 + June 2018	0,396727	0,092964	0,140147	0,434187
June 2018 + September 2018	0,789675	0,454889	0,826091	0,872118
Muscles -T-test between measurements	Girls	Boys	Mild ID	Moderate ID
in each category	p	p	p	p
June 2017 + September 2017	1,000000	0,656642	0,798860	0,278708
September 2017 + June 2018	0,820280	0,940481	0,084285	0,433049
June 2018 + September 2018	0,426529	0,454889	**0,041328**	0,064150
Balance R- leg -T-test between measurements	Girls	Boys	Mild ID	Moderate ID
in each category	p	p	p	p
June 2017 + September 2017	0,674424	0,593712	0,201244	0,674987
September 2017 + June 2018	0,833635	0,139757	**0,017291**	0,982627
June 2018 + September 2018	0,308064	0,660282	0,058025	0,981117
Balance L- leg -T-test between measurements	Girls	Boys	Mild ID	Moderate ID
in each category	p	p	p	p
June 2017 + September 2017	0,722283	0,656642	0,833936	0,694887
September 2017 + June 2018	0,838464	0,162674	0,109746	0,958761
June 2018 + September 2018	0,109746	0,849817	0,153577	0,727538

I highlighted statistically significant differences at the 5% level of significance.

In the muscle category, there is a statistically significant difference only between the last measurements (p = 0,041328) in pupils with mild ID. There is also a statistically significant difference in the balance of the R-leg in the middle of the observed period for pupils with mild ID (p = 0,017291). For the other categories, there are no statistically significant differences in the muscles and postural balance.

### 3.4 A comparison and correlation of postural balance with muscles

In the mild ID, the improvement in the balance on both the L and R leg (for 30 s in June 2017 and 2018) is related to the muscle growth (23 kg in June 2017 and 23.45 kg in June 2018), (Table 9).

**Table 9:** A Comparison of postural balance with muscles.

Category	Balance - median (sec)		Muscles - median (kg)
Mild ID	R-leg - mild ID	L-leg - mild ID	Muscles - mild ID
June 2017	**30,00**	**30,00**	**23,00**
September 2017	26,00	25,50	20,70
June 2018	**30,00**	**30,00**	**23,45**
September 2018	28,00	27,00	18,85
Moderate ID	R-leg - moderate ID	L-leg - moderate ID	Muscles - moderate ID
June 2017	**5,10**	**5,50**	13,00
September 2017	**5,00**	5,00	**19,30**
June 2018	4,00	**6,00**	17,75
September 2018	3,00	5,00	**19,60**
Girls	R-leg - moderate ID	L-leg - moderate ID	Muscles - girls
June 2017	**26,50**	**17,00**	**19,40**
September 2017	**16,00**	**18,00**	**19,30**
June 2018	15,00	13,00	18,30
September 2018	3,00	5,00	18,50
Boys	R-leg - moderate ID	L-leg - moderate ID	Muscles - boys
June 2017	**16,00**	**12,00**	21,20
September 2017	7,50	5,50	**21,70**
June 2018	**25,00**	**13,00**	**24,20**
September 2018	12,00	11,00	20,30

Highlighting of the highest values in balance and muscles.

Also in girls, the improvement in the balance of the L and R leg is associated with an increase in muscle mass. But in the moderate ID and in boys, there is no clear correlation between improved balance and muscle growth.

The following table (Table 10) shows the correlation between postural balance and muscles.

**Table 10:** Correlation between postural balance and muscles in each category.

Spearman correlation coefficient	Balance							
	June 2017		September 2017		June 2018		September 2018	
p < 0,05	R - leg	L - leg	R - leg	L - leg	R - leg	L - leg	R - leg	L - leg
Muscles girls	0,392	0,290	-0,050	0,087	0,259	0,136	-0,161	-0,093
Muscles boys	**0,752**	**0,744**	0,038	0,335	-0,050	-0,031	-0,196	-0,195
Muscles mild ID	0,372	-0,009	**0,740**	**0,502**	-0,146	-0,047	-0,049	0,101
Muscles moderate ID	0,162	0,358	0,053	0,025	-0,107	-0,182	-0,226	-0,389

I highlighted the stronger value of the correlation.

There is a positive strong correlation between the balance on the R-leg and L-leg and muscles in boys in June 2017 (R-leg r = 0.752 and L-leg r = 0.744) and in pupils with mild ID in September 2017 (R-leg r = 0.740 and L-leg r = 0.502). This means that improved balance is directly proportional to increased muscles (that is, the better their balance, the more muscles they have).

However, the positive correlation is based on half of the cases. In the other half of the cases, there is a negative weak correlation, indicating the opposite (improvement in balance indicates muscles loss).

## 4 Discussion

It is quite surprising to find that boys and girls, as well as pupils with mild or moderate intellectual disability, achieve, in terms of median number of steps per day, far greater than 50 % of the recommended number of steps per day and that the values are indeed close to the norm (girls reach 86–97 %, boys 89–109 %). Many authors agree that people with intellectual disability have lower than the recommended level of daily physical activity (Foley et al., 2008; Peterson et al., 2008; Frey et al., 2008; Shields et al., 2009; Einarsson et al., 2015; Boddy et al., 2015) and according to most of them, their physical activity is about half the norm.

The results of physical activity of children in the Czech Republic are much higher than what is stated by other authors (Boddy et al., 2015; Einarsson et al., 2015; Shields et al., 2009; Wouters et al., 2018). Compared to the number of steps, Phillips and Holland (2011) report slightly higher physical activity in boys of 7 181 ± 179 steps per day than in girls of 6 918 ± 749 steps per day. Izquierdo-Gomez et al. (2014) also found higher physical activity in boys than in girls with intellectual disability. In our research, boys have results of 13 092 ± 7 174; 11 957 ± 5 771; 10 650 ± 6 495; 11 987 ± 5 793 steps per day and girls have 11 662 ± 5 159; 11 595 ± 5 371; 10 272 ± 5 965; 12 132 ± 6 177 steps per day. Thus, boys had greater physical activity than girls in three of the four measurements. In the last measurement, the girls had higher physical activity levels. The outcome may be influenced by the greater motor skills of boys with intellectual disability than girls with intellectual disability, as reported (Rintala and Loovis, 2013; Simons et al., 2008; Westendorp et al., 2014).

The patterns of physical activity during adolescence tend to be carried into adulthood. Thus, active children can be expected to become active adults. This has benefits for public health (Telama et al., 2005; Hallal et al., 2006; Telama 2009). From this point of view, the trend of physical activity in children and adolescents with ID in the Czech Republic is going in the right direction and the current situation, both in education and in services and opportunities for sports activities available to children with ID - should be maintained.

In terms of weekly physical activity, it is interesting to note that of all the evaluated categories, pupils with mild intellectual disability have the most PA both in terms of the median number of steps per day (101–122 % of the norm), and in terms of the number of people (50–69 % of all pupils with mild ID have sufficient physical activity of 12 000 steps per day and more every day). By comparison, in the UK, only 23 % of children and adolescents have recommended daily physical activity (Boddy et al., 2015). In Australia, 42 % of children and adolescents with mild intellectual disability (Shields et al., 2009) and in the Netherlands, 47 % of children and adolescents have recommended daily PA (Wouters et al., 2018).

The high physical activity of pupils with mild intellectual disability is probably because their degree of disability does not limit them in movement as much as pupils with moderate intellectual disability. This is confirmed by Švarcová (2000, pp. 28–29), who reports large individual differences, less ability to move and be independent in pupils with moderate intellectual disability. We also presume that pupils with mild intellectual disability make much greater use of sport clubs in their schools and that they are more interested in exercise. We note that these children always had reduced physical activity, lower BMI values and muscle loss during the summer holidays compared to the school year. We see from this the importance of primary school and the activities it offers to pupils during the year: clubs (sports, gymnastics), a ski course, an outdoor school, road safety training, swimming lessons, participation in SO competitions (a ski course, a floorball tournament, a soccer tournament, an athletic Olympiad). The school therefore has a positive effect on the pupils' physical activity. Furthermore, we see that the impact of ČHSO events, which are organized for children with intellectual disability during the school year, is significant. The school regularly participates in these events with pupils, and this was probably reflected in the high physical activity of the pupils. Pupils at schools are also cared for by qualified teachers, so the results are robust. Common educational activities in schools include the development of motor skills, the development of self-care and personal hygiene. In the training “apartment”, children learn to prepare simple meals, wash up, iron, etc. The influence of school on pupils is in short fundamental.

In children and adolescents with moderate ID there is an increase in their physical activity and muscle growth following the summer holidays and we could deduce from this that parents spend far more time with their children during the holidays than during the school year.

Weight gain is more common in people with ID (Melville et al., 2007; Rimmer and Yamaki, 2006). Women with ID are more prone to obesity than men with ID. The risk of obesity is also high in people with Down syndrome and mild ID (Melville et al., 2007; Hsieh et al., 2014). This contrasts with our research, where in terms of BMI values for all monitored groups - boys, girls, pupils with mild ID and pupils with moderate ID are in the category of normal weight throughout the 2-year research. In terms of muscle development trends, pupils with mild ID had more muscles in 3 of 4 measurements than pupils with moderate ID. We can therefore conclude that pupils with mild ID have more movement than pupils with moderate ID.

Interestingly, there was no clear correlation (positive or negative) in the postural balance and pupils' muscles. This may be due to the children having their eyes open during the test and fixing their gaze to help keep their balance. According to Kolář (2009), the balance is affected by the weight, the height of the center of gravity and the area of the support base. Visual nerve pathways, vestibular system, cardiovascular activity, respiration, auditory stimulation, drugs, standing length, height, gender also have an effect (Kapteyn et al., 1983). We can therefore summarize that there are many individual factors affecting each person tested, and therefore there was probably no clear correlation.

The limitation of this research is the relatively small number of participants and the region. Considering the system of special needs schools across the country we could assume that the offer of school clubs for children with intellectual disabilities is similar in most schools, and it is therefore possible to assume similar results in physical activity levels of children and adolescents in other schools in the Czech Republic. It appears that in the Czech Republic children have comparatively far more opportunities to participate in physical activities than in other countries.

## 5 Conclusions

In girls, the trend of physical activity and the trend of muscles is convex, the trend of BMI is unbalanced. Summer holidays cause a decrease in BMI values for girls. Girls achieve 86–97 % of the norm of physical activity. Their BMI is in the normal weight category.

In boys, the trend of physical activity is convex and the trend of BMI is unbalanced. The muscle trend is concave. Summer holidays cause a decrease in BMI values for boys. Boys reach 89–109 % of the norm of physical activity. Their BMI is in the normal weight category.

In in children and adolescents with mild ID, the trend of physical activity is unbalanced, the trend of BMI and the trend of muscles is also unbalanced. Summer holidays cause a decrease in physical activity, a decrease in BMI values and muscle loss. Children and adolescents with mild ID achieve 101–122 % of the norm of physical activity (as the only category in each measurement they are above the norm). Their BMI is in the normal weight category.

In in children and adolescents with moderate ID, the trend of physical activity is unbalanced, the trend of BMI and the trend of muscles is also unbalanced. Summer holidays cause an increase in physical activity and an increase in muscle (both changes are statistically significant). Furthermore, summer holidays cause a decrease in BMI values. Pupils with moderate ID reach 74–99 % of the norm of physical activity. Their BMI is in the normal weight category. Wilcoxon's t-test found statistically significant differences between all successive measurements of physical activity in children and adolescents with moderate ID.

The correlation between physical activity and BMI and muscles in girls and boys is ambiguous. The values of the correlation coefficient range from a negative mean correlation to a positive weak correlation.

The correlation between physical activity and BMI and muscles in children and adolescents with mild and moderate ID is also ambiguous. The values of the correlation coefficient range from a negative strong correlation to a positive mean correlation.

Improvement in postural balance in connection with muscle growth was demonstrated in girls and pupils with mild ID at the end of the school year in June.

The correlation between postural balance and muscles is ambiguous in girls, in boys, in pupils with mild ID and with moderate ID. The values of the correlation coefficient range from a negative mean correlation to a positive strong correlation. A positive strong correlation between postural balance and muscles was demonstrated in only one measurement in boys and in pupils with mild ID.

It is important for the development of the best practice in the Czech Republic to find that the physical activity of children and adolescents with intellectual disability is at a good level. Children with ID have far more exercise than children with ID in other states. For the Czech Republic, the research findings are an indicator that the education for children with intellectual disability is well established and works well. The cooperation of schools with ČHSO also works well, as children have a lot of opportunities to participate in sport events during the year. It is therefore recommended that the current trend of education and cooperation with ČHSO continues.

## Declarations

### Author contribution statement

Hana Válkova: Conceived and designed the experiments; Performed the experiments; Contributed reagents, materials, analysis tools or data.

Jitka Kampasová: Performed the experiments; Analyzed and interpreted the data; Contributed reagents, materials, analysis tools or data; Wrote the paper.

### Funding statement

This research did not receive any specific grant from funding agencies in the public, commercial, or not-for-profit sectors.

### Data availability statement

Data will be made available on request.

### Declaration of interests statement

The authors declare no conflict of interest.

### Additional information

No additional information is available for this paper.
